# Pexidartinib and standard neoadjuvant therapy in the adaptively randomized I-SPY2 trial for early breast cancer

**DOI:** 10.1007/s10549-024-07555-9

**Published:** 2024-12-03

**Authors:** Hope S. Rugo, Mike Campbell, Christina Yau, A. Jo Chien, Anne M. Wallace, Claudine Isaacs, Judy C. Boughey, Hyo S. Han, Meredith Buxton, Julia L. Clennell, Smita M. Asare, Katherine Steeg, Amy Wilson, Ruby Singhrao, Jeffrey B. Matthews, Jane Perlmutter, W. Fraser Symmans, Nola M. Hylton, Angela M. DeMichele, Douglas Yee, Laura J. Van’t Veer, Donald A. Berry, Laura J. Esserman

**Affiliations:** 1 University of California San Francisco, Box 1710, San Francisco, CA 94143 USA; 2 University of California San Diego, San Diego, CA USA; 3 Georgetown University, Washington, DC USA; 4 Mayo Clinic, Rochester, MN USA; 5 Moffitt Cancer Center, Tampa, FL USA; 6 Quantum Leap Healthcare Collaborative, San Francisco, CA USA; 7 Gemini Group, Ann Arbor, MI USA; 8 University of Texas MD Anderson Cancer Center, Houston, TX USA; 9 University of Pennsylvania, Philadelphia, PA USA; 10 University of Minnesota, Minneapolis, MN USA; 11 Berry Consultants, LLC, Houston, TX USA

**Keywords:** Breast cancer, Clinical trial, Colony-stimulating factor-1, Pexidartinib

## Abstract

**Purpose:**

We investigated the small-molecule receptor tyrosine kinase-inhibitor of colony-stimulating factor-1 receptor pexidartinib in the stage II/III breast cancer in the I-SPY2 platform trial.

**Methods:**

I-SPY2 is an adaptive platform trial that features multiple arms of experimental agents administered on a background of standard neoadjuvant therapy with paclitaxel and adriamycin/cyclophosphamide, followed by definitive surgery. The adaptive randomization engine preferentially assigns patients based upon cumulative performance of each agent in a given breast cancer subtype based on hormone receptor and HER2 receptor status. The study endpoint is pathologic complete response.

**Results:**

A total of 9 participants were randomized to receive pexidartinib with neoadjuvant paclitaxel before enrollment was halted due to a serious adverse event of vanishing bile duct syndrome. No participants received a full course of the study drug.

**Conclusion:**

Although there remains interest in agents targeting CSF-1, hepatic toxicity appears to be a limiting factor for their use in early breast cancer.

**Trial registration:**

NCT01042379 (www.clinicaltrials.gov/ct2/show/NCT01042379).

## Introduction

Colony-stimulating factor-1 (CSF1) is a key cytokine involved in recruitment and activation of tissue macrophages [1]. CSF1R is overexpressed or mutated in breast, as well as other cancer types, correlating with disease progression and malignancy [2]. In cancer, CSF-1R signaling facilitates the recruitment and survival of tumor associated macrophages (TAMs) in the tumor microenvironment, resulting in suppression of host anti-tumor immunity [3] The interaction between macrophages and tumor immunity is complex, with both pro- and anti-tumor subsets comprising the tumor infiltrate; macrophages promote tumorigenesis and metastases by numerous mechanisms, including secreting cytokines that enhance tumor proliferation and angiogenesis [4]. Inhibition of CSF-1 with tyrosine kinase inhibitors or antibodies are one strategy to inhibit or deplete the pro-tumor M2 macrophage subset [5, 6].

Pexidartinib (PLX3397, Daiichi-Sankyo) is a small-molecule receptor tyrosine kinase-inhibitor of CSF1 receptor (CSF1R), FMS-like tyrosine kinase 3 (FLT3) and stromal cell factor receptor (Kit) [7, 8], and is approved for the treatment of adults with symptomatic tenosynovial giant cell tumor (TGCT) associated with severe morbidity or functional limitations and not amenable to improvement with surgery. The rationale for combining pexidartinib and paclitaxel for the treatment of advanced solid tumors comes from In vivo studies in the polyoma middle T (PymT) mouse breast cancer model demonstrating that the combination reduced macrophage infiltration, reduced tumor growth, and reduced the occurrence of pulmonary metastases compared with paclitaxel alone [5].

Based on this evidence, we evaluated pexidartinib together with paclitaxel as neoadjuvant therapy for early breast cancer at high risk of recurrence in the I-SPY2 phase II adaptive platform trial.

## Patients and methods

### Study design

I-SPY2 is a phase II, multicenter, adaptive, platform trial of neoadjuvant therapy for early breast cancer at high risk of recurrence, where multiple experimental agents are evaluated in parallel against a common control arm [9, 10]. Pathologic complete response (pCR) is the primary endpoint. Additional details of the study design have been published previously [9, 10].

### Eligibility

I-SPY2 is open to adults aged 18 or older with stage II/III breast cancer, with tumor > 2.5 cm by clinical exam or > 2 cm by imaging and molecularly high-risk disease if HR-positive. All participants signed written informed consent.

### Treatment and assessments

All patients received standard of care, consisting of twelve weekly doses of intravenous paclitaxel 80 mg/m^2^, followed by 4 cycles of chemotherapy (AC) consisting of intravenous doxorubicin 60 mg/m^2^ and cyclophosphamide 600 mg/m^2^ every 2–3 (per physician discretion) weeks.

Patients randomized to the pexidartinib experimental arm received oral pexidartinib at a dose of 1200 mg daily during the 12 weeks of paclitaxel. Following AC, participants underwent definitive surgery, with lumpectomy or mastectomy at the discretion of the treating surgeon. Serial breast MRI was used to assess response as previously described [9, 10].

### Trial oversight

The trial was designed by I-SPY2 investigators. Plexxikon (now part of Daiichi-Sankyo) provided study drug but played no role in the study design, collection/analysis of data or in manuscript preparation. All participating sites received institutional review board approval and a DSMB met monthly to review patient safety and study progress. The authors of the manuscript vouch for the accuracy and completeness of the data reported.

### Statistical analysis

Standard I-SPY standard statistical approaches have been published previously, but were not applicable given the early termination of the arm.

## Results

### Patients

**Fig. 1:**
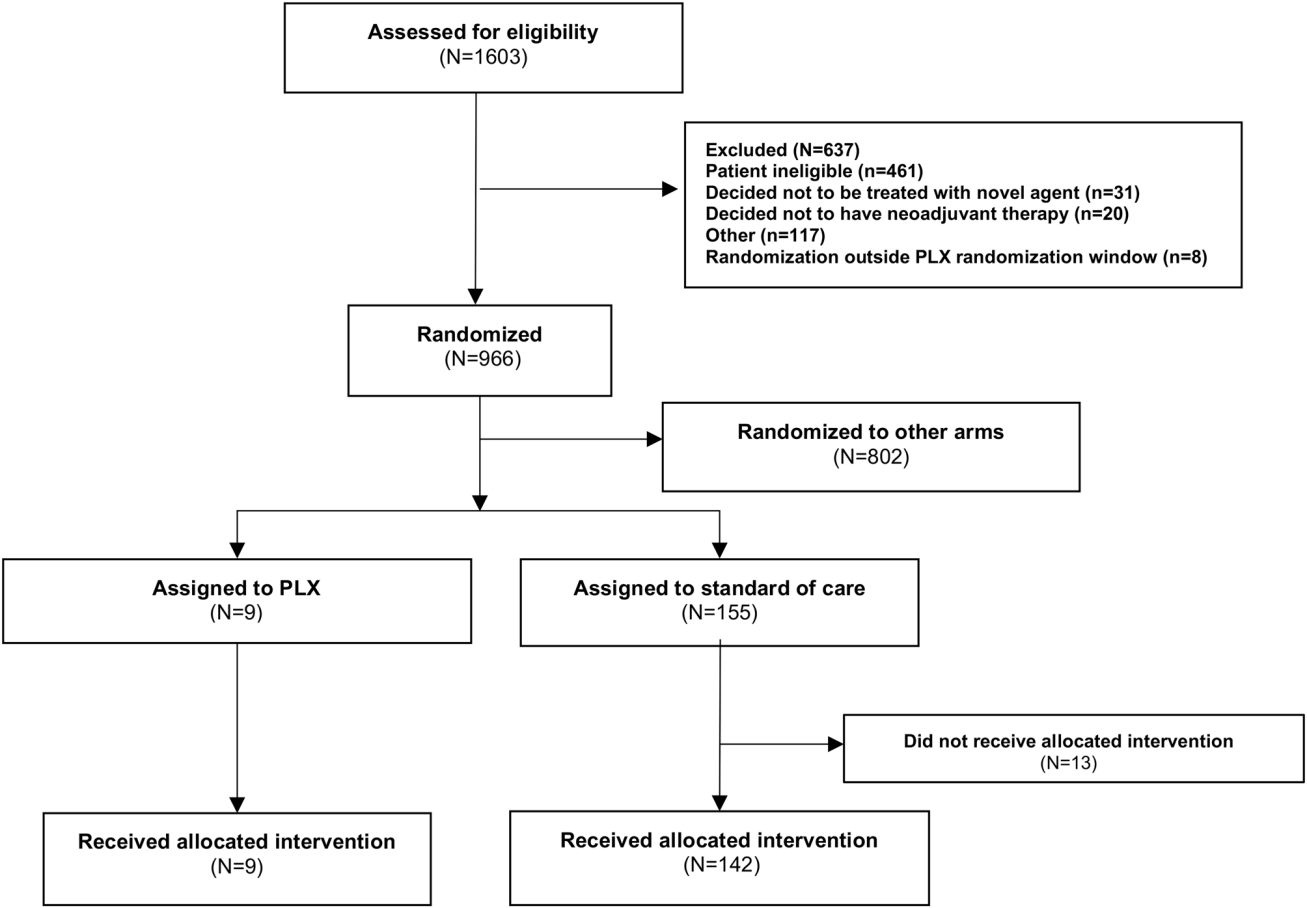
Consort diagram including patients randomized to the Pexidartinib/paclitaxel and control arms

The pexidartinib arm opened for enrollment on 17 August 2015. A total of 9 participants were eligible and began treatment in the experimental arm. On 22 October 2015, enrollment to the arm was halted following the report of a serious adverse event (SAE) in a patient three weeks after initiation of therapy. The control arm population, per-protocol, consisted of 142 participants enrolled between 3 March 2010 and the date the pexidartinib arm was closed to enrollment (Fig. 1).

All participants were female, with HER2-negative disease; 7 were hormone receptor (HR)-positive/HER2-negative, 2 were triple negative (TN). Five of the nine participants in the pexidartinib arm had evidence of axillary node involvement, and mean pre-treatment tumor diameter was 5.98 cm by MRI. Average age was 44.8 yr. in the pexidartinib arm, compared to 48.1 in control subjects. Other baseline characteristics are shown in Table 1.

**Table 1:** Baseline characteristics

	PLX3397 (*n* = 9)	Control (*n* = 142)
Age	44.8 (33–60)	48.1(24–77)
Race/ethnicity		
Hispanic or Latino	0 (0%)	16 (11%)
Not Hispanic or Latino	9 (100%)	126 (89%)
Asian	0 (0%)	7 (5%)
Asian; White	0 (0%)	2 (1%)
Black or African American	1 (11%)	22 (15%)
Native Hawaiian or Pacific Isl	0 (0%)	0 (0%)
White	8 (89%)	111 (78%)
Subtypes		
HR−/HER2−	2 (22%)	69 (49%)
HR+/HER2−	7 (78%)	73 (51%)
MRI (longest diam., cm)	5.98 (2.2–15.0)	4.80 (1.2–15.0)
Palpable nodes		
NA	0 (0%)	11 (8%)
No	4 (44%)	64 (45%)
Yes	5 (56%)	67 (47%)

### Safety & toxicity

Of the nine patients receiving pexidartinib/paclitaxel, all received at least one dose of experimental agent. One patient was treated with dose-reduced pexidartinib and four participants discontinued pexidartinib treatment due to toxicity; the remaining 5 participants discontinued pexidartinib therapy when the arm closed. No participants received the full course of study drug (Table 2); two completed AC and 7 completed surgery on protocol.

**Table 2:** Grade 3/4 adverse events observed in experimental and control arms during each phase of treatment—paclitaxel ± pexidartinib and doxorubicin/cyclophosphamide

Adverse event		Pexidartinib (*n* = 9)		Control (*n* = 142)	
		Paclitaxel + pexidartinib (*n* = 9)	AC (*n* = 0)	Paclitaxel (*n* = 142)	AC (*n* = 116)
Face oedema		1 (11.1%)	–	–	–
Cholecystitis		1 (11.1%)	–	–	–
Hepatobiliary disease		1 (11.1%)	–	–	–
Hepatotoxicity		1 (11.1%)	–	–	–
Liver disorder		1 (11.1%)	–	–	–
Anaphylactic reaction		1 (11.1%)	–	–	–
Alanine aminotransferase increased		3 (33.3%)	–	2 (1.4%)	–
Aspartate aminotransferase increased		2 (22.2%)	–	1 (0.7%)	–
Blood alkaline phosphatase increased		1 (11.1%)	–	–	–
Blood bilirubin increased		1 (11.1%)	–	–	–
Neutrophil count decreased		3 (33.3%)	–	8 (5.6%)	4 (3.4%)
White blood cell count decreased		1 (11.1%)	–	4 (2.8%)	3 (2.6%)
Dehydration		1 (11.1%)	–	–	2 (1.7%)
Arthralgia		2 (22.2%)	–	1 (0.7%)	–
Back pain		1 (11.1%)	–	1 (0.7%)	–
Myalgia		1 (11.1%)	–	1 (0.7%)	–
Syncope		2 (22.2%)	–	–	2 (1.7%)
Rash maculo-papular		2 (22.2%)	–	–	–
Toxicity					
Dose reductions, *n* (%)	1 (11.1%)		–	4 (2.8%)	8 (6.9%)
Early discontinuation, *n* (%)					
All	9 (100%)		–	26 (18.3%)	7 (6.0%)
Toxicity	4 (44.4%)		–	7 (4.9%)	4 (3.4%)
Progression	–		–	5 (3.5%)	1 (0.9%)
Other	5 (55.6%)		–	14 (9.9%)	2 (1.7%)
Time from consent to surgery (days)					
Median (range)	164 (68–225)			165 (100–289)	

Five patients had grade 3/4 liver enzyme elevation; additional grade 3/4 AE are shown in Table 2 and supplement. There was one SAE, documented in a published case report, after 3 weeks of pexidartinib/paclitaxel therapy; the patient presented with fever of > 39 °C with markedly elevated liver function tests including total bilirubin and study therapy was discontinued [11]. Following exhaustive workup, a diagnosis of acute drug-induced liver injury, suggestive of vanishing bile duct syndrome (VBDS) was made, attributed as likely due to the pexidartinib/paclitaxel combination. Following multiple interventions over the course of 36 months, including liver transplant, the patient’s liver function tests and performance status improved significantly. She remained disease free from breast cancer on adjuvant letrozole at last follow-up in 2/2019.

### Response and long-term outcomes of patients on the PLX arm

Subjects receiving experimental therapy at the time of arm closure were considered non-evaluable. Due to closure of the pexidartinib arm, we did not perform the standard I-SPY2 primary efficacy analysis comparing the pexidartinib arm against controls. Instead, we provide a summary of response rates and event-free survival (EFS) of pexidartinib-treated patients. Of the 9 patients on pexidartinib (2 TN and 7 HR-positive/HER2-negative), one switched to carboplatin with continued paclitaxel, 3 switched to paclitaxel, one to nab-paclitaxel, and 2 patients withdrew consent prior to surgery and were considered non-pathologic complete response (pCR) per-protocol. One TN patient achieved pCR with standard therapy and one patient with HR-positive/HER2-negative disease had minimal residual disease (residual cancer burden, RCB-I). The other five HR-positive/HER2-negative patients had intermediate or extensive residual tumor burden (4 RCB-II, 1 RCB-III); two of these 5 patients experienced a distant recurrence.

## Discussion

Pexidartinib was granted breakthrough therapy designation by the U.S. Food and Drug Administration (FDA) in 2015 for TGCT based on phase 1 results showing significant response [12]. Final FDA approval in 2019 was based on positive results from the phase III ENLIVEN trial [7, 13].

Studies have reported elevated aminotransferases as the most common adverse effect of pexidartinib (seen in approximately 50% of patients), generally considered to be a consequence of CSF-1 pathway inhibition in Kupffer cells in the liver; as well as evidence of cholestatic hepatotoxicity [7, 12–14]. A long-term study of pooled hepatic safety data in TGCT reported 95% of patients experiencing an hepatic AE, although no patients met criteria for Hy’s law. Out of 658 patients treated for non-TGCT indications, 2 experienced irreversible hepatic AEs. Of note, this data had not been reported at the time of our trial [15]. Other reported toxicities include hair color changes, periorbital edema, vomiting, fatigue, and dysgeusia [13].

For this study, pexidartinib dose was selected based on safety and efficacy data from the phase 1b trial of pexidartinib and weekly paclitaxel in patients with advanced solid tumors [12]. There, the pexidartinib/paclitaxel combination was well tolerated, with no dose-limiting or high-grade hepatic toxicity reported [12]. However, hepatic toxicity in treatment naïve patients resulted in early closure of this I-SPY2 arm, with one case of life-threatening toxicity (VBDS) requiring hepatic transplantation [11]. It is difficult to evaluate efficacy given limited exposure to the combination therapy. However, given the very low rate of response in this small study population it is reasonable to include that short-term exposure to pexidartinib did not enhance response to paclitaxel in patients with high-risk early-stage HER2-negative breast cancer.

Additional studies have evaluated CSF-1 inhibitors in breast cancer [16, 17]. Kuemmel et al. randomized 49 patients with advanced TN disease and high levels of tumor-associated macrophages to receive gemcitabine and carboplatin with/without the anti-CSF-1 antibody lacnotuzumab [17]. There was no difference in progression-free survival between the arms, although the study was closed early, and liver enzyme elevation was seen in > 80%, with half ≥ grade 3. The anti-CSF-1 antibody LY302285 (IMC-CS4) also resulted in liver enzyme elevations and minimal efficacy in a phase I dose-escalation trial in solid tumors [18], and minimal toxicity in a second study in patients with refractory breast and prostate cancer, with a best response of stable disease [16]. This drug has also been evaluated in melanoma and pancreatic cancer without reported results.

The potential to modify the macrophage component of tumor-infiltrating lymphocytes and enhance the anti-tumor host immune response remains intriguing, but not at the cost of the severe toxicity we observed. Hepatic toxicity appears to be an on-target toxicity of CSF-1 antagonists. Newer, approved immune-oncology strategies using checkpoint inhibitors with less toxicity have since shown dramatic improvements to outcomes. Other approaches to depleting tumor-associated macrophages may be possible, such as antibodies against triggering receptor expressed on myeloid cells 2 (TREM2), or TREM1 to reprogram of immunosuppressive myeloid cells [19, 20], though safety concerns will have to be addressed before this class of agents progresses.

## Data Availability

De-identified subject level data are available to members of the research community by approval of the I-SPY Data Access and Publications Committee. Details of the application and review process are available at https://www.quantumleaphealth.org/for-investigators/clinicians-proposal-submissions/.
